# The association of mannose binding lectin genotype and immune response to *Chlamydia pneumoniae*: The Strong Heart Study

**DOI:** 10.1371/journal.pone.0210640

**Published:** 2019-01-10

**Authors:** Laine Monsey, Lyle G. Best, Jianhui Zhu, Susan DeCroo, Matthew Z. Anderson

**Affiliations:** 1 Department of Microbiology, The Ohio State University, Columbus, OH, United States of America; 2 Missouri Breaks Industries Research Inc., Timber Lakes, SD, United States of America; 3 Medstar Research Institute, Washington, DC, United States of America; 4 Department of Microbial Infection and Immunity, The Ohio State University, Columbus, OH, United States of America; Public Health England, UNITED KINGDOM

## Abstract

Cardiovascular disease (CVD) is an important contributor to morbidity and mortality in American Indian communities. The Strong Heart Study (SHS) was initiated in response to the need for population based estimates of cardiovascular disease in American Indians. Previous studies within SHS have identified correlations between heart disease and deficiencies in mannose binding lectin (MBL), a motif recognition molecule of the innate immune system. MBL mediates the immune response to invading pathogens including *Chlamydia pneumoniae* (*Cp*), which has also been associated with the development and progression of CVD. However, a link between *MBL2* genotype and *Cp* in contributing to heart disease has not been established. To address this, SHS collected baseline *Cp* antibody titers (IgA and IgG) and *MBL2* genotypes for common functional variants from 553 individuals among twelve participating tribes. A single nucleotide polymorphism (SNP) in the promoter, designated X/Y, correlated significantly with increased *Cp* IgG titer levels, whereas another promoter SNP (H/L) did not significantly influence antibody levels to *Cp*. Two variants within exon 1 of *MBL2*, the A and B alleles, also displayed significant association with *Cp* antibody titers. Some *MBL2* genotypes were absent from the population, suggesting linkage disequilibrium may be operating within the SHS cohort. Additional factors, such as increasing age and socioeconomic status, were also associated with increased *Cp* IgG antibody titers. This study demonstrates that *MBL2* genotype associates with immune reactivity to *C*. *pneumoniae* in the SHS cohort. Thus, *MBL2* may contribute to the progression of cardiovascular disease (CVD) among American Indians indirectly through pathogen interactions in addition to its previously defined roles.

## Introduction

Cardiovascular disease contributes substantially to poor global health. In the United States, cardiovascular disease (CVD) is the leading cause of death, responsible for 1 in 4 deaths [1]. Although decreasing considerably over three decades starting in the 1980's, total mortality rate due to CVD increased by 3% between 2011 and 2014 and has been consistently higher than cancer-related deaths [2]. Clinical manifestations include damage to the epithelial lining of blood vessels, inflammation of the heart or arterial lining; and eventually restricted blood flow, which results in myocardial infarction, stroke, and numerous other manifestations

Cardiovascular disease risk is largely composed of two primary sources, environmental contributions and genetic predisposition. Environmental contributors to CVD include diet, lifestyle, air quality, and infectious agents [3]. The relative contributions of these factors vary across time, geography, and socioeconomic status. Consequently, environmental factors shape the impact of CVD on specific populations differently. Some factors such as infectious disease outbreaks and air quality are communal and require large-scale concerted efforts to combat while diet and lifestyle include individualized components. Preventative therapies exist that alleviate the impact of personalized risks such as smoking cessation programs and lifestyle/dietary intervention [4] but do not address factors that impact whole populations. For example, previous work has linked locally endemic infectious agents to CVD risk including HIV, *Trypanasoma cruzi*, and *Chlamydia pneumoniae*, which are highly prevalent within particular global regions [5, 6].

Cardiovascular disease is a complex genetic disease influenced by the contribution of thousands of variant positions across hundreds of loci [7]. Immunomodulatory molecules comprise a significant portion of genes implicated in CVD and include genes such as C-reactive protein (*CRP*), apolipoprotein E (apoE), and mannose binding lectin (*MBL2*) [8–10]. MBL is a major circulating C-type lectin of the innate immune system involved in self versus non-self recognition. As such, MBL plays a significant role in identification of dead, dying, or cancerous cells for clearance by professional phagocytes [11]. Similarly, MBL binds to a variety of pathogen-associated molecular patterns (PAMPs) leading to pathogen recognition and destruction [12, 13]. *MBL2* dysregulation is associated with coronary heart disease (CHD) and increased susceptibility to microbial infection [14, 15].

One of the pathogens most consistently associated with CVD onset and progression is *Chlamydia pneumoniae* (*Cp*), an obligate intracellular bacterium that infects humans, and specifically targets endothelial cells [6, 16–19]. *C*. *pneumoniae* is capable of producing both active, acute disease as well as latent infections that persist throughout the life of the host with the potential to re-emerge. During active infection, accumulation of cellular debris during rupture of infected cells as well as attraction of circulating phagocytes can lead to plaque formation on blood vessel walls, inducing vascular complications such as coronary heart disease (CHD) [6]. *MBL2* operates as a recognition receptor for mannose and N-acetylglocusamine, which are major components of the *Cp* cell membrane. Importantly, reduced MBL function has been linked to CVD severity and progression in patients infected with *Chlamydia pneumoniae* [17, 20, 21].

Variants within the *MBL2* coding sequence and promoter region, (see S1 Table), have significant consequences on its molecular function. Several well characterized *MBL2* promoter and coding missense SNPs significantly alter circulating levels of MBL and are associated with clinical disease [22, 23]. The frequency of these coding alleles within groups varies depending on population origin. For example, the B allele frequency is approximately 45% in South American Mapuche and Chiriguano communities, whereas it is present in less than 1% of a Mozambican population [24, 25]. Such population-based variation in allele frequency may contribute to differences in disease risk among specific demographics.

The Strong Heart Study (SHS) is the largest epidemiological study within American Indian populations and is focused on cardiovascular health [26]. Twelve tribes have participated in the Strong Heart Study to develop reliable, population-based data related to cardiovascular health among American Indians. Previous studies within the SHS cohort identified an association between *MBL2* genotypes and coronary artery disease [27]. However, the relationship between the immune response to *Cp* and *MBL2* genotypes associated with CVD has not yet been established.

Here we investigated the relationship between *Cp* and *MBL2* within the SHS cohort. *MBL2* promoter and coding sequence genotype frequencies do not reflect majority Caucasian populations. *Cp* antibody titers are significantly affected by the X/Y promoter variant and the copy number of *MBL2 ‘*A’ and ‘B’ alleles. Several additional descriptive factors correlate with *Cp* antibody levels including age and education. Thus, development of CVD may be due, in part, to the interaction of the host immune system with infectious agents such as *Cp*.

## Methods

### Participants

This ancillary study was conducted between August 1, 2000, and November 30, 2000, among individuals enrolled in the Strong Heart Study (SHS) at ages 45 to 74 years between July 1, 1989, and January 31, 1992. It was approved by the Indian Health Service, MedStar Research Institute Institutional Review Boards, and by the participating tribes. All investigative methods conformed to the principles set out by the Declaration of Helsinki and involved informed consent of the participants. Since these data were collected, one tribe has declined further participation and those tribal members have been excluded from the analysis. All studies are reviewed and approved by the SHS Review Board composed of tribal representatives prior to study initiation and publication.

The original study’s design and methods have been reported [26]. An ancillary study of the relationship between infectious disease and CVD measured baseline antibodies to C pneumoniae and other pathogens on a subset of 421 CVD cases and an equal number of controls, matched by study center, sex and age (+/- 5 years). From this substudy, *MBL2* genotypes were available from 553 individuals. Medical record review by trained medical record abstractors and a physician reviewer committee determined that 211 had experienced definite CVD, 161 possible CVD and 181 no CVD in subsequent follow-up. Risk factors for CVD were determined at time of entry into the SHS and at two subsequent follow-up examinations.

### Blood specimen collections

Blood specimens collected during the study were separated into serum samples, frozen at -80°C, and aliquots were thawed only when performing specific tests.

### *MBL2* locus genotyping

Genotyping was done at the University of Pittsburgh. A total of 553 individuals were assessed for the presence of the *MBL2* B allele (G54D, rs1800450), C allele (G57E, rs1800451) or D allele (R52C, rs5030737) structural variations, and two promoter polymorphisms, one a G/C transition at—550 bp (the H/L alleles, rs11003125) and the other, a G/C transition at -221 (X/Y alleles, rs7096206). The most common coding allele has been conventionally labelled "A" and the three structural variants collectively labelled "O". *MBL2* genotypes were determined by the oligonucleotide ligation assay as described by Nickerson and colleagues [28]. Quality control, duplicate, genotyping was performed by direct DNA sequencing. The structural variations were assumed to occur on opposing chromosomes. A number of promoter variants and structural alleles have been found to be in complete linkage disequilibrium and genotypes were checked against these established relationships [28].

*MBL2* allele frequencies for comparisons were obtained from the following studies: Dutch [29], Inuit [30], Mozambican [25], South Korean [31], and those from the United States [32].

Haplotypes were built using previously described methods [27]. Briefly, homozygous positions could be inherently linked to alleles at other variant positions. Subsequently, unresolved haplotypes were built using previously described association including the B and C coding alleles and X promoter variant being found with the L promoter variant, the D *MBL2* allele linked to the H promoter variant, etc.,

### *Cp* antibody titer

Serum IgG and IgA antibody titers for *C*. *pneumoniae* were determined by microimmunofluorescence (MIF) at the International Chlamydia Laboratory at Johns Hopkins School of Medicine. This test uses the application of purified elementary bodies (EBs) from high titer chlamydia EB antigen preparations (Washington Research Foundation, Seattle, WA). There is only one serovar of *C*. *pneumoniae* and the purified antigen is made from strain AR39. Antigen dots for *C*. *trachomatis* were also be included in the series of antigen dots, so that the specificity of the anti-*C*. *pneumoniae* antibody could be confirmed. The highest dilution of serum demonstrating good even fluorescence of the EBs was recorded as the titer for each group. Separate assays were performed for the determination of IgG, and IgA. The laboratory has participated with others in quality assurance studies for MIF and demonstrated consistency and comparability for this assay. The *Cp* antibody titers could be determined for 514 individuals.

### Statistical analysis

Statistical testing for correlational analysis were performed through SPSS and R (v3.3.1) software. SPSS was developed by Microsoft, and R was developed by the R development team.

## Results

**Fig 1:**
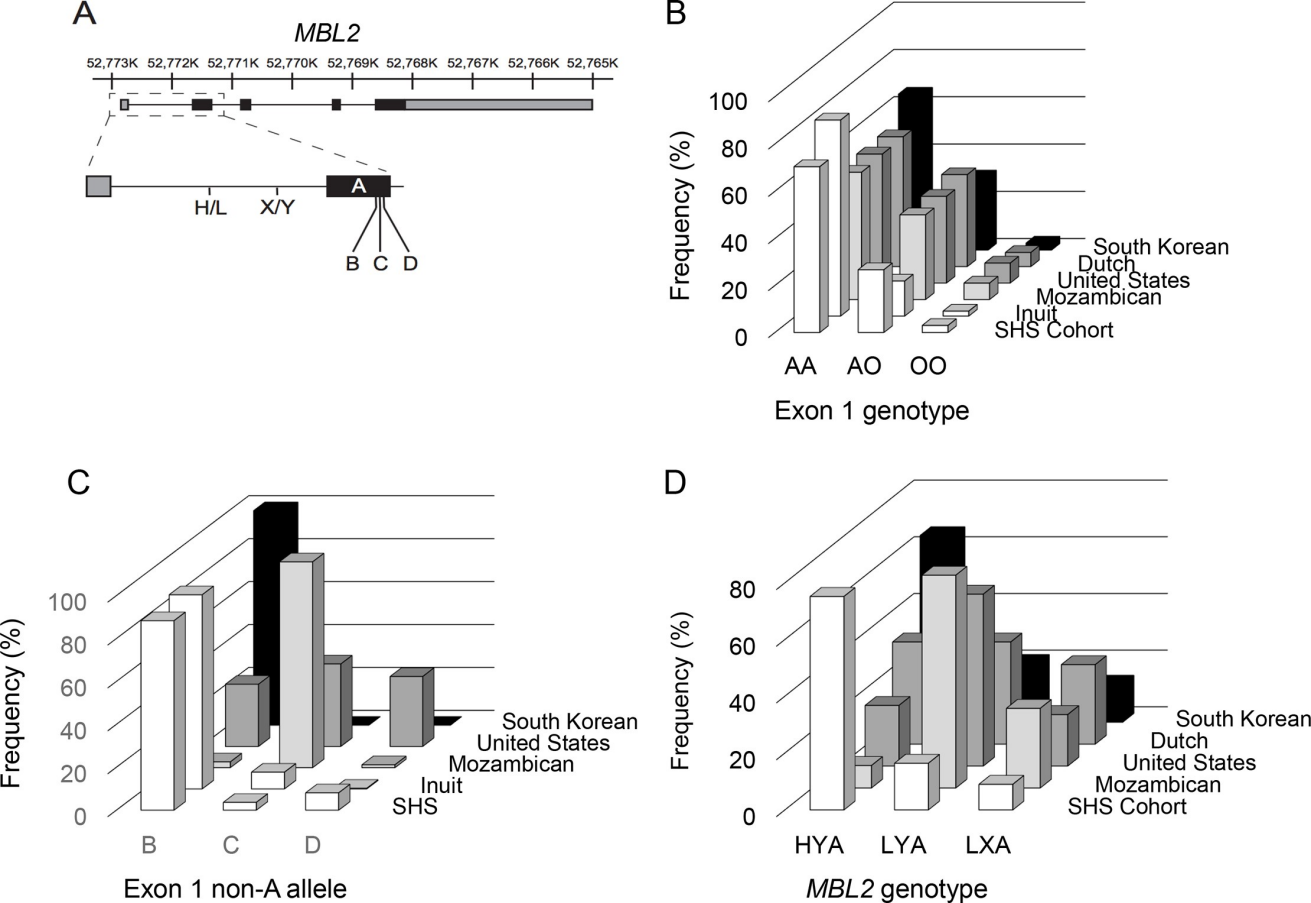
Genotype frequency of *MBL2* among the SHS cohort. **A.** The *MBL2* gene is composed of 5 exons comprised of both noncoding (grey) and coding (black) sequences. Two prominent polymorphic sites associated with *MBL2* function exist in the promoter at positions -550 (H/L variant) and -221 (X/Y variant). Additionally, the genotype of exon 1 impacts gene function based on three variant alleles B, C, and D corresponding to amino acid changes at codon 52, 54, and 57, respectively, as well as the canonical allele A. The frequency of exon 1 genotypes for allele A (**B**), non-A alleles (O) (**C),** or promoter variants (**D**) was plotted for five previously reported populations and the SHS cohort. The number of individuals plotted are: SHS (553), Dutch (212), Inuit (148), Mozambiquan (185), and Korean (128).

The *MBL2* gene is a key component of innate immunity whose product recognizes pathogen-associated molecular patterns of invading organisms and marks them for destruction. The functional capacity of *MBL2* is associated with five polymorphic sites, two in the promoter and three within the first exon [33]. The two promoter variant positions, denoted as H/L and X/Y, are located 550 and 221 nucleotides upstream of the start codon, respectively (Fig 1A). Polymorphisms at these positions impact the level of *MBL2* transcription with the H and Y variants producing more robust expression [28]. Additionally, variants of exon 1 referred to as alleles B, C, and D encode changes at amino acid 52, 54, and 57 compared to the reference A allele. The B and C alleles produce proteins with reduced function due to a decrease in oligomerization capability required for function and the D allele is especially weakened in its immunological role [28].

### *MBL2* genotypes and distributions

Previous investigations within the Strong Heart Study cohort identified links between heart disease and low functioning *MBL2* genotypes [17, 27]. To determine the distribution of *MBL2* alleles within the SHS cohort using an expanded panel of polymorphic positions, individuals were genotyped at both promoter polymorphic sites and exon 1. The majority of individuals encoded the functional A/A *MBL2* genotype (69.6%) with a smaller subset either heterozygous or homozygous for a "non-A" (O) allele (27.5% and 2.9%, respectively) (Table 1). The B variant was the most common O allele (14.3%) followed by the D and C alelles (1.6% and 0.7%, respectively).

**Table 1:** Frequency of *MBL2* genotypes within the SHS cohort.

SNP position	Homozygous (Major)	Heterozygous	Homozygous (Minor)
H/L	0.467 (HH)	0.389 (HL)	0.14 (LL)
X/Y	0.888 (YY)	0.11 (XY)	0.003 (XX)
A	0.696 (AA)	0.275 (AO)	0.029 (OO)
B		0.252 (B(A/O))	0.029 (BB)
C		0.007 (C(A/O))	0.00 (CC)
D		0.016 (D(A/O))	0.00 (DD)

Similar exon 1 allele frequencies are observed among distinct populations from different geographic regions for the A allele (Fig 1B). In contrast, the distribution of non-A alleles within exon 1 differed widely among distinct populations. The SHS cohort, Inuit, and South Korean populations harboured high frequencies of allele B, the Mozambican population primarily contained C allele variants, and the United States population had an even distribution among all three non-A alleles (Fig 1C). Furthermore, promoter alleles frequencies varied considerably among representative populations (Fig 1D). In the SHS cohort population the H and Y variants comprised 66.2% and 94.3% of the population, respectively (Table 1). Alleles within all genotyped variant positions conformed to Hardy-Weinberg equilibrium (S2 and S3 Tables). The SHS cohort was most similar to the Inuit and South Korean populations, suggesting that the frequency of *MBL2* genotypes among American Indians significantly differs from Americans of European descent.

*MBL2* haplotypes were constructed from allelic genotypes to assess association of promoter and coding variants. Haplotype construction revealed a majority of HYA haplotypes among SHS participants associated with robust Mbl2 function (Table 2). Thus, relatively few haplotypes containing L variants emerged from construction and X variant haplotypes were very rare. Consequently, all assembled haplotypes included at least one high function allele from either the promoter or exon 1.

**Table 2:** Frequency of *MBL2* haplotypes.

Population	HYA	LYA	LXA
Dutch [29]	0.36	0.36	0.28
Inuit [30]	0.89	0.06	0.05
Mozambican [25]	0.08	0.75	0.17
SHS cohort	0.75	0.16	0.09
South Korean [31]	0.66	0.21	0.13
United States [32]	0.39	0.29	0.33

Comparison to *MBL2* haplotypes from other global populations most resembled the Inuit and South Korean populations, similar to analysis of individual variant positions.

### Association of *MBL2* genotype with *C. pneumoniae*

**Fig 2:**
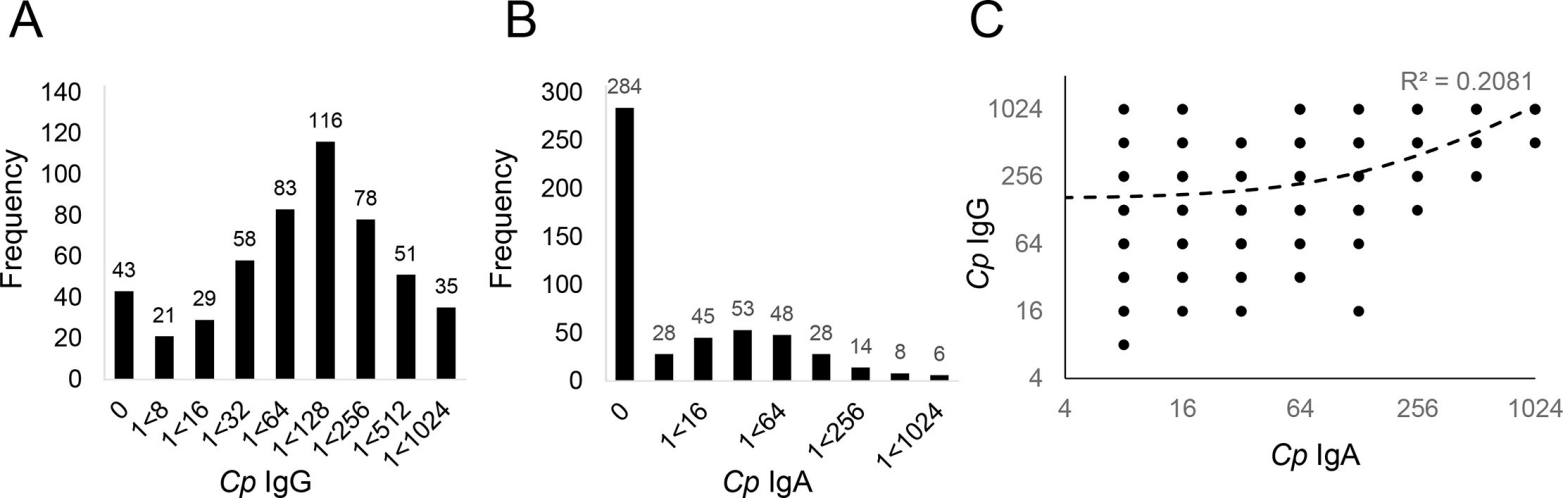
Antibody titers to *C*. *pneumoniae* are correlated. The *C*. *pneumoniae* antibody titer of 514 individuals within the SHS cohort was plotted for IgG (**A**) or IgA (**B**). **C.** Antibody titers for IgG and IgA are significantly correlated within individuals (Spearman, r_s_ = 0.47, *p*<0.01).

To test for an association between *MBL2* genotype and immune reactivity to *C*. *pneumoniae*, antibody titers were measured for systemic IgG and mucosa-enriched IgA. *C*. *pneumoniae* IgG titers followed a Gaussian distribution centered on 1:128, indicating relatively strong systemic reactivity (Fig 2A). Participants expressed lower levels of IgA to *C*. *pneumoniae*, however, with more than half exhibiting no detectable antibody (Fig 2B). A positive linear correlation exists between *Cp* IgG and IgA within the SHS cohort (Spearman, r_s_ = 0.47, *p*<0.01, Fig 2C). Consequently, the majority of our subsequent analysis relied upon IgG antibody titers as an indicator of immune reactivity.

**Fig 3:**
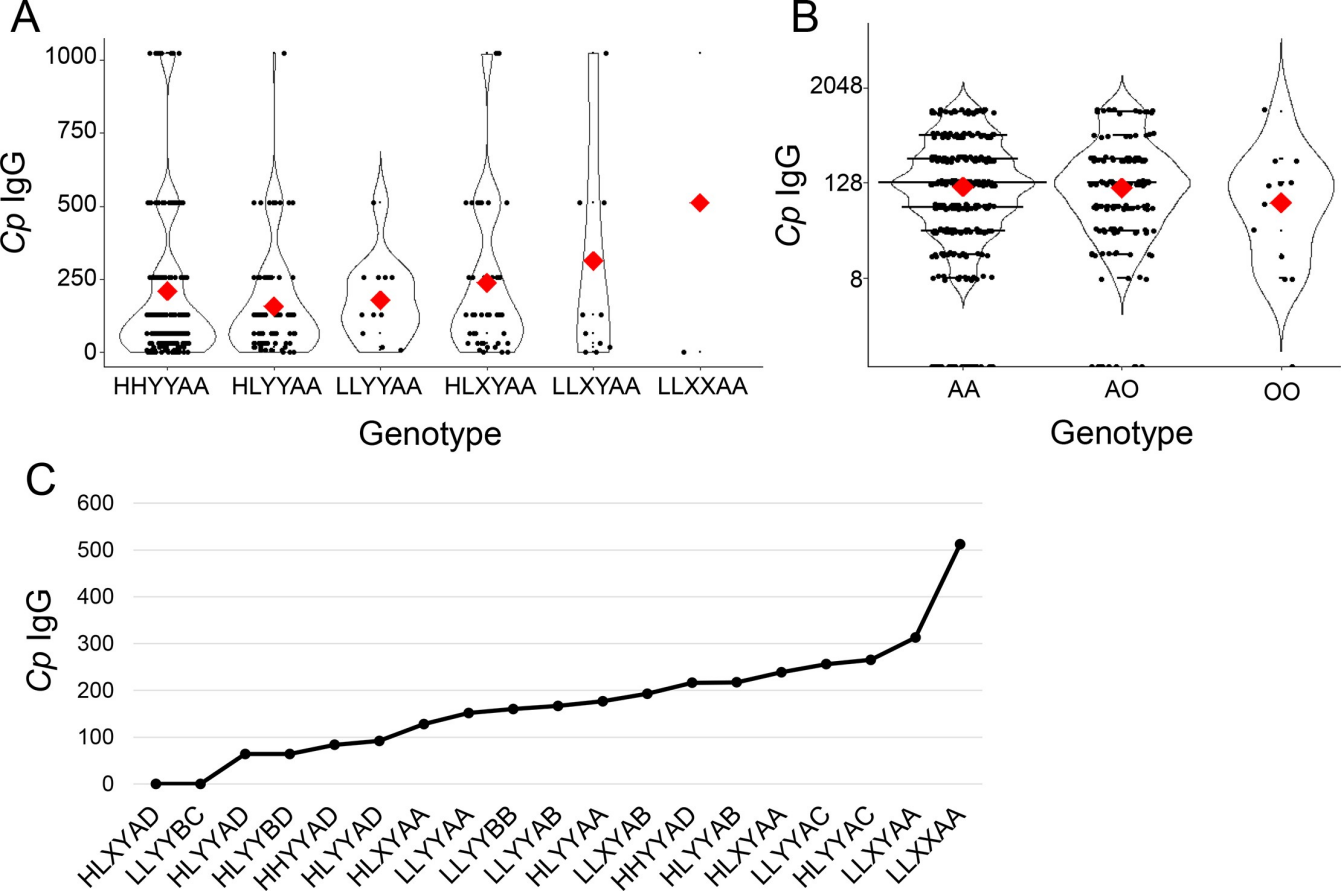
Polymorphic sites within *MBL2 are* associated with altered *C*. *pneumoniae* antibody titers. **A.** The mean *C*. *pneumoniae* antibody titer (red) is significantly correlated with *MBL2* promoter genotypes (F(1,644) = 3.895, p = 0.049). Increased copies of the X (X/Y) variant resulted in elevated antibody titers among these 514 genotyped individuals. **B.** Genotyping of 514 participants indicated the presence of the canonical A allele of exon 1 was associated with increased *C*. *pneumoniae* antibody titer (F(1,644) = 4.39, p = 0.037). The mean is denoted in red. **C.** The mean antibody titer for all 553 *MBL2* genotypes within the SHS cohort increases with elevated copy number for the H, X, and A alleles.

To determine the influence of *MBL2* polymorphism on *Cp* antibody titers, we assessed the distribution of antibody levels for individual SNP positions. Analysis of covariance revealed additional copies of the X allele are significantly correlated with elevated *Cp* IgG titers (F(1,544) = 3.895, p = 0.049, Fig 3A, Table 3).

**Table 3:** ANCOVA of *Cp* IgG versus *MBL2* promoter SNP positions.

Source	dF	F	MSE	Partial Eta Squared	p-value
L allele	1	1.934	647188.321	0.003	0.165
Y allele	1	3.895	134039.451	0.006	0.049

The H/L polymorphic site trended towards increased *Cp* antibody titers with increased dosage of the L allele but did not reach significance (F(1, 544) = 1.934, p = 0.165, Fig 3A).

The coding variants of exon 1 also impacted antibody levels to *C*. *pneumoniae*, *with Cp* antibody titer increasing with each additional A allele (F(1,544) = 4.39, p = 0.037, Fig 3B, Table 4). The B allele was also predictive of *Cp* IgG antibody titers (F(1,544) = 4.48, p = 0.035, Table 4). Neither the C nor D allele displayed any association with *Cp* titers although this may be due, in part, to their low prevalence and hence, lack of power. Taken together, both promoter and coding sequence alleles correlate with *Cp* antibody titers in the SHS cohort.

**Table 4:** ANCOVA of *Cp* IgG versus *MBL2* exon 1 alleles.

Source	dF	F	MSE	Partial Eta Squared	p-value
A allele	1	4.389	303623.570	0.000	0.037
B allele	1	4.479	309858.252	0.007	0.035
C allele	1	2.802	193877.528	0.004	0.095
D allele	1	1.863	129062.322	0.003	0.173

The aggregate contributions of individual polymorphic sites to *Cp* antibody titers can either be additive or show more complex interactions. To investigate this, the average *Cp* IgG titers were plotted for each represented genotype in the SHS cohort (Fig 3C, S1 Fig). No specific position determined the relative antibody levels within the cohort, suggesting that the combined effects of each variant contribute to the overall immune reactivity. For example, the *MBL2* A and L alleles were enriched among higher *Cp* antibody titer groups and co-occurred in individuals with the highest average antibody titers. Additionally, the X promoter variant clustered with genotypes displaying higher Ab titers. Interestingly, the reported strength of *MBL2* function for variant positions did not seem consistent with *Cp* antibody titers; the high-functioning *MBL2* A allele corresponded to increased antibody levels, while promoter variants with reduced function (X and L) also correlated with higher *Cp* antibody titers. Across variant positions, the *MBL2* A allele displayed the strongest association with *Cp* antibody titers (S2 Fig). Furthermore, analysis of constructed haplotypes including promoter and coding variants also failed to identify a significant association with *Cp* antibody titer (F(6, 1094) = 1.575, p = 0.151). Thus, individual promoter and coding sequence variants but not complete genotypes correlate with antibody production to *Cp*.

### Associations of covariates

**Fig 4:**
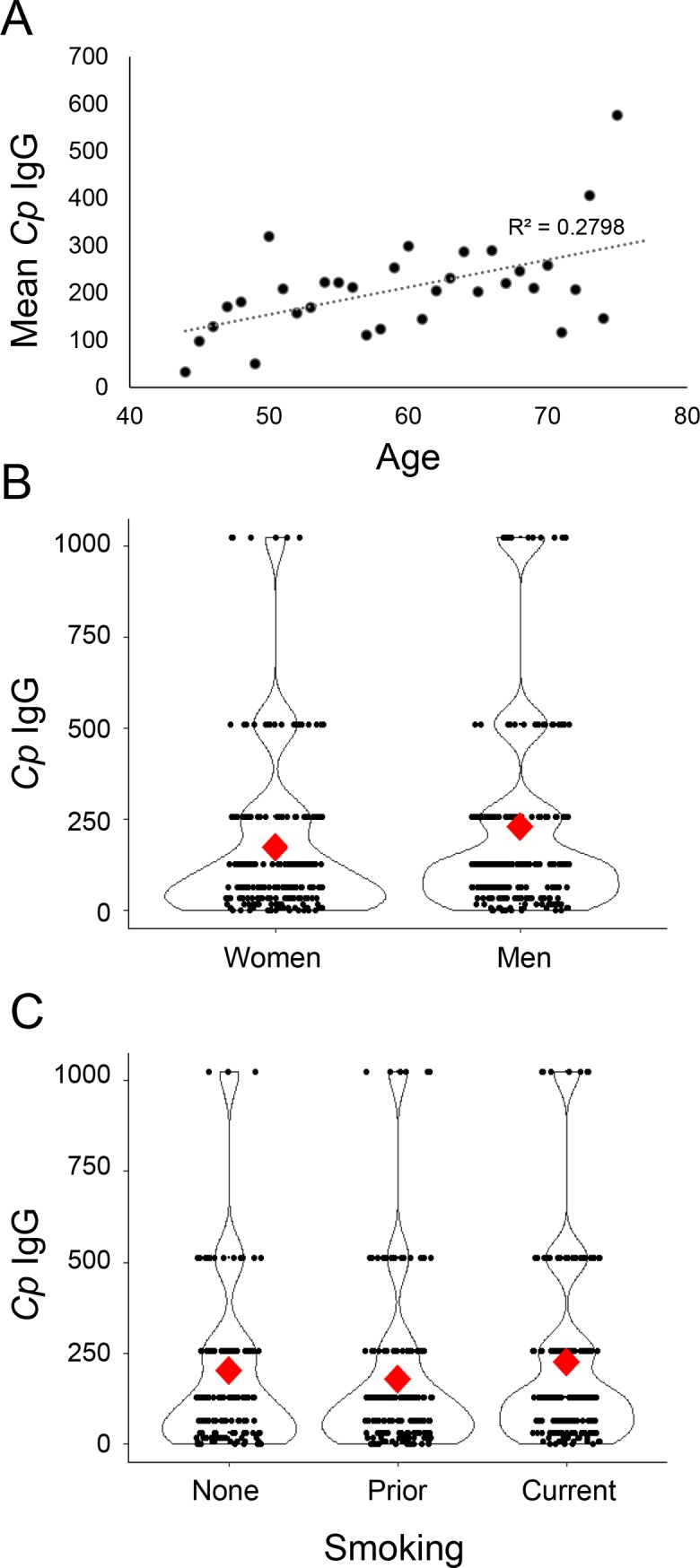
Age, gender, and education are associated with *C*. *pneumoniae* antibody titer. **A.** The mean *C*. *pneumoniae* antibody titer increased for each represented age within the SHS cohort (Spearman’s correlation; *r*_*s*_ = 0.09 p < 0.041). **B.** The mean *C*. *pneumoniae* antibody titer (red) was elevated for men compared to women (Mann Whitney; U = 3.27, p = 0.001). N = 514 individuals. **C.** The mean *C*. *pneumoniae* antibody titer (red) increased among current smokers compared to non-smokers or previous smokers (Kruskal Willis; H(2) = 7.185, *p* = 0.028). N = 514 individuals.

Changes in *Cp* antibody titers can be influenced by multiple non-genetic factors that are produced by lifestyle and the environment [34]. We tested the association of *Cp* IgG titers against 14 variables including age, sex, education, smoking, alcohol use, ACR, and prior diagnoses of cardio vascular disease and diabetes (S4 Table). Three features showed significant associations with *Cp* IgG antibody levels, age (Spearman’s correlation; *r*_*s*_ = 0.09 p < 0.041), smoking (Kruskal Willis; H(2) = 7.185, *p* = 0.028), and sex (Mann Whitney; U = 3.27, p = 0.001) (Fig 4). Additionally, the presence of the Y or H variants in the *MBL2* promoter strongly correlated with a prior diagnoses of diabetes (*r*_*s*_ = -0.106, .111; *p* = <0.01 for both) (S4 Table).

### Association of *Cp* with CVD

*Cp* infection has been previously associated with autoimmune disorders including CVD [17, 20]. To test for this association, participant genotypes were separated based on CVD diagnosis as either present, possible, or absent. No relationship between CVD and *Cp* antibody titers existed among SHS participants with diagnosed CVD versus healthy individuals (Mann-Whitney U-test (MW); F(1,369) = 1.135, p = 0.256, S4 Table). Furthermore, a test for association between *Cp* antibody titers and mortality due to CVD failed to identify a relationship (MW; F(1,369) = 0.879, p = 0.379). However, the H/L *MBL2* promoter variant associated with CVD in which increased copies of low expression L alleles associated with increased CVD diagnosis as would be expected (S4 Table). Thus, no clear relationship between immune reactivity to *Cp* and CVD within this SHS population can be established.

## Discussion

Here, we have shown that *MBL2* genotypes within an American Indian cohort correlated with the immunological response to the intracellular bacteria *C*. *pneumoniae*. *MBL2* promoter variants within the SHS cohort were not found at similar frequencies compared to majority Caucasian populations. Both *MBL2* promoter and coding variants were associated with the prevalence of antibody following exposure to *C*. *pneumoniae*, suggesting differences in immunological response although constructed haplotypes diminished the associations of individual variants with *Cp* antibody titers. The degree of immunogenicity was also correlated with more complex social and biological processes such as aging and education. These factors may contribute to the elevated risk of CVD within Native American communities.

Participants reported here had unique *MBL2* genotype frequencies consistent with another Indigenous population in North America and similar to East Asian populations [25, 30, 31, 35, 36]. While the SHS prevalence of the majority A allele of *MBL2* is similar to but slightly elevated compared most global populations, the B allele was much more common compared to populations of majority European or African ancestry [25, 32, 37]. Furthermore, the distribution of promoter variants and haplotypes in the SHS cohort differed markedly from the majority Caucasian population in the US [32, 38] and most closely resembled an Inuit and Korean population [30, 31]. Increased prevalence of high expression H and Y promoter variants in the SHS cohort could significantly influence the response to pathogens through *MBL2*, as well as impact other, intrinsic immunological functions that may contribute to chronic inflammation and hence influence CVD among American Indian populations.

Within the SHS cohort, all allelic variants were present but some specific genotypes were missing. Combinations of *MBL2* “C” and “D” alleles were present only with “A” or “B” despite being found together in similarly sized cohorts within the US [32]. Additionally, certain promoter genotypes were absent from the population despite being present in combination with other alleles (i.e., HHXX). Initial studies of *MBL2* haplotypes also reported an absence of the HX haplotype [39]; however, recent studies have described its presence among a number of distinct global populations [40, 41]. Interestingly, none of these reports include Indigenous peoples of the Americas, suggesting that these genotypes may be absent because of maintained haplotype blocks that do not recombine or are selected against when present. It is tempting to speculate that selective pressures have modulated MBL activity by altering genotypic frequencies, reflecting the dual roles of controlling infectious agents and promoting cardiovascular function.

MBL2 functions as a “first responder” within the innate immune system in recognizing pathogens such as *C*. *pneumoniae*, which have been implicated in promoting CVD. Two particular *MBL2* variant positions correlated with antibody production to *Cp*, the X/Y promoter variant and exon 1. Surprisingly, the high functioning A allele of *MBL2* correlated with elevated *Cp* antibody titers; in a consistent manner, the lower functioning B allele correlated with reduced *Cp* antibody titers. The high prevalence of the *MBL2* B allele and its corresponding reduced function may play a role in the development of CVD within American Indian populations specifically [14]. Within the promoter, both low expression X and L alleles were associated with increased antibody production. Similarly, immunization of mice lacking *MBL2* led to increased IgG production compared to wildtype mice [42]. It is unclear how *MBL2* variants that promote opposing levels of expression and/or function both increase *Cp* antibody titers but suggests that production of antibody through MBL regulation is complex, which is further supported by the non-additive nature of individual alleles in haplotype construction with immune reactivity. Additionally, a large prospective study demonstrated the unexpected relationship between high prevalence of *MBL2* genotypes associated with high expression and increased risk of CVD [43]. Thus, additional factors such as Interleukin-6 (IL-6) and C-reactive protein (CRP), which contribute to *Cp* antibody production, and other host-specific factors may contribute to these discrepancies [44].

Other environmental factors contributed significantly to *Cp* antibody titers among the SHS cohort. Our data indicated a linear relationship between age and higher amounts of circulating *Cp* antibody. This trend is consistent with previous studies that found an increase in seroprevalence transitioning from childhood into adulthood [34, 45, 46] and extends those studies to suggest that immune response to *Cp* continues to accumulate later in life as well. Association between *Cp* antibody titers and smoking have also been noted previously although the mechanism is unclear [46]. The impact of gender on *Cp* immune reactivity is less well defined as studies have found both higher antibody titers in males as we observed [47] or no difference between sexes [46], suggesting this may vary depending on the sampled population.

The previously observed association between *MBL2* genotype and CVD may include both the direct action of MBL in regulation of host cellular maintenance and its additional role in immune surveillance of pathogens [27]. For example, some *MBL2* variants correlated with increased levels of *Cp* antibodies were also shown to associate with CVD risk[27]. Thus, *MBL2* genotyping could be used in the clinic to screen individuals for host- and microbe-associated risk factors in CVD development. As a result of screening, physicians could introduce preventative strategies earlier to reduce the likelihood of disease onset and progression within American Indians populations.

## Supporting information

S1 Fig *C*. *pneumoniae* antibody titers for SHS cohort genotypes. The *C*. *pneumoniae* antibody titer for IgG (**A**) or IgA (**B**) are plotted for all genotypes among the 553 individuals found within the SHS cohort.
(TIF)

S2 Fig Association of promoter and coding allelic variants are associated in *MBL2*. The relationship between *MBL2* genetic variants and *Cp* antibody titers was constructed using hierarchical clustering and Euclidean distances.
(TIF)

S1 Table Nomenclature of studied *MBL2* variant positions. (TIF)

S2 Table *MBL2* allele frequencies in the SHS cohort conformed to the Hardy-Weinberg equilibrium. (TIF)

S3 Table *MBL2* Exon 1 frequencies in the SHS cohort conformed to the Hardy-Weinberg equilibrium. (TIF)

S4 Table Correlation analysis of genotypic and phenotypic traits. Pairwise associations of traits were performed using a Spearman’s correlation analysis for all genetic polymorphisms assayed and a number of important covariates. Pink indicates p<0.05 and red indicates p<0.01.
(TIF)
