# Supplementary figures and images for: The association of mannose binding lectin genotype and immune response to Chlamydia pneumoniae: The Strong Heart Study
Source: PLoS One. 2019 Jan 10;14(1):e0210640. doi: 10.1371/journal.pone.0210640 (PMC6328205; doi:10.1371/journal.pone.0210640)

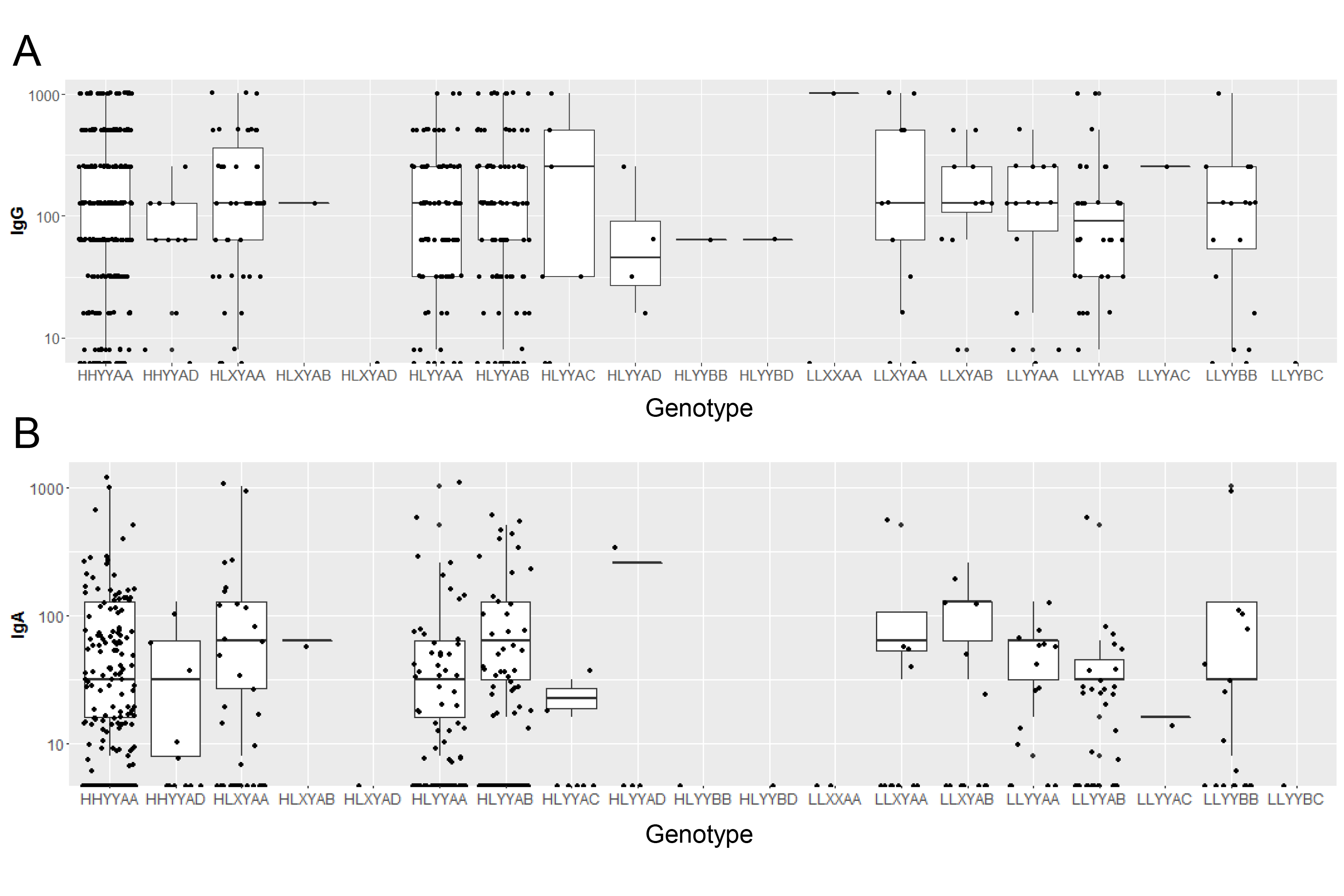

Supplement: S1 Fig — The C. pneumoniae antibody titer for IgG (A) or IgA (B) are plotted for all genotypes among the 553 individuals found within the SHS cohort. (TIF) [file pone.0210640.s001.tif]

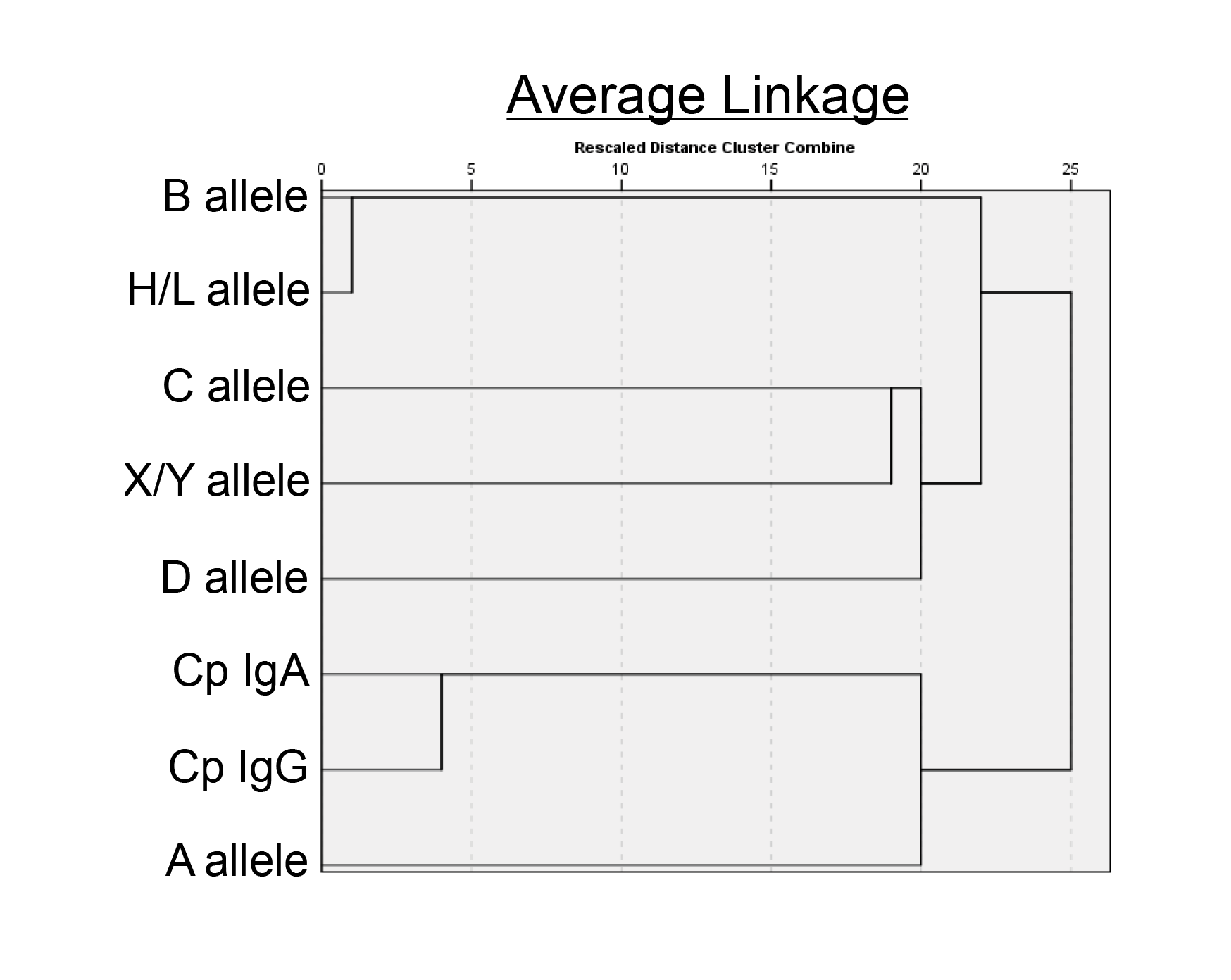

Supplement: S2 Fig — The relationship between MBL2 genetic variants and Cp antibody titers was constructed using hierarchical clustering and Euclidean distances. (TIF) [file pone.0210640.s002.tif]

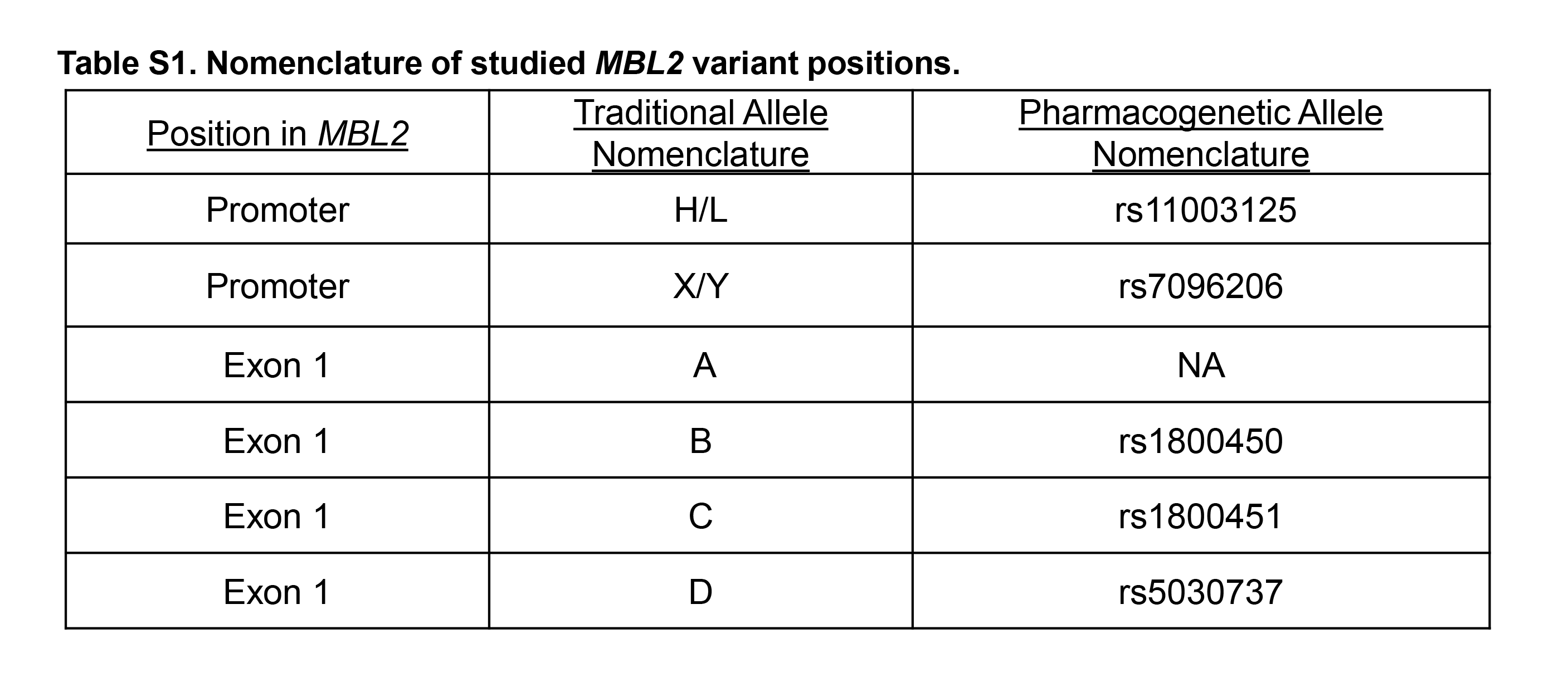

Supplement: S1 Table — (TIF) [file pone.0210640.s003.tif]

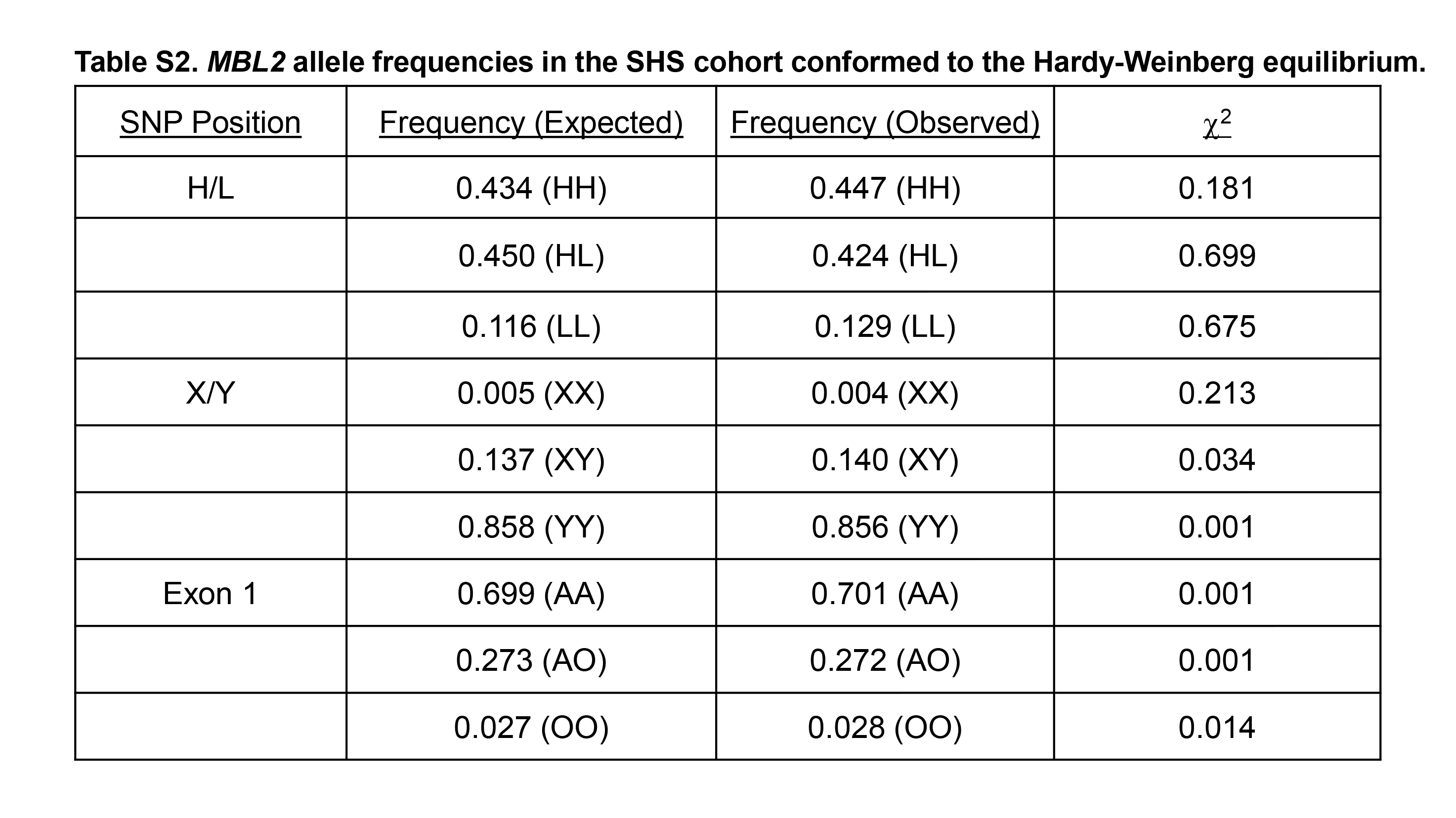

Supplement: S2 Table — (TIF) [file pone.0210640.s004.tif]

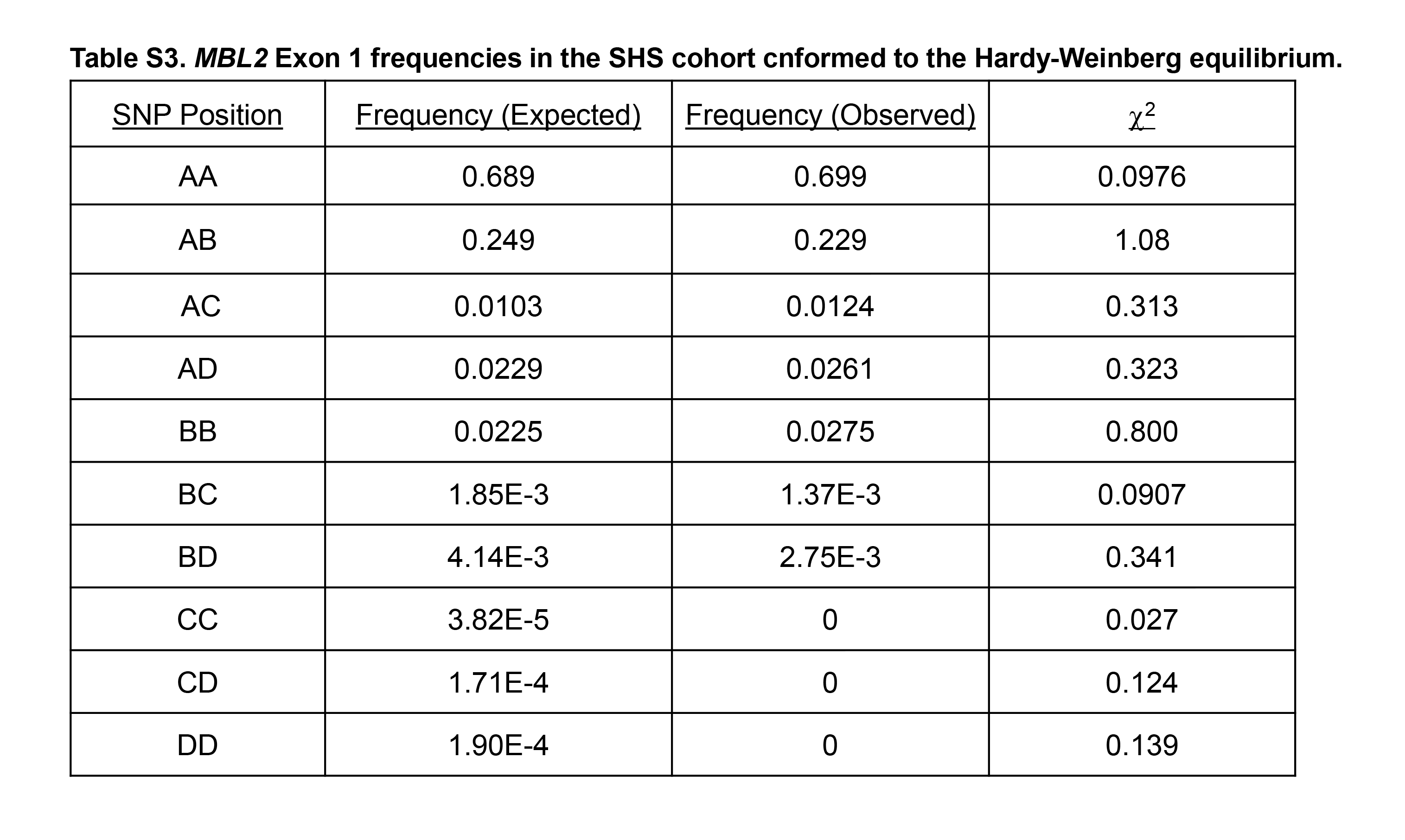

Supplement: S3 Table — (TIF) [file pone.0210640.s005.tif]

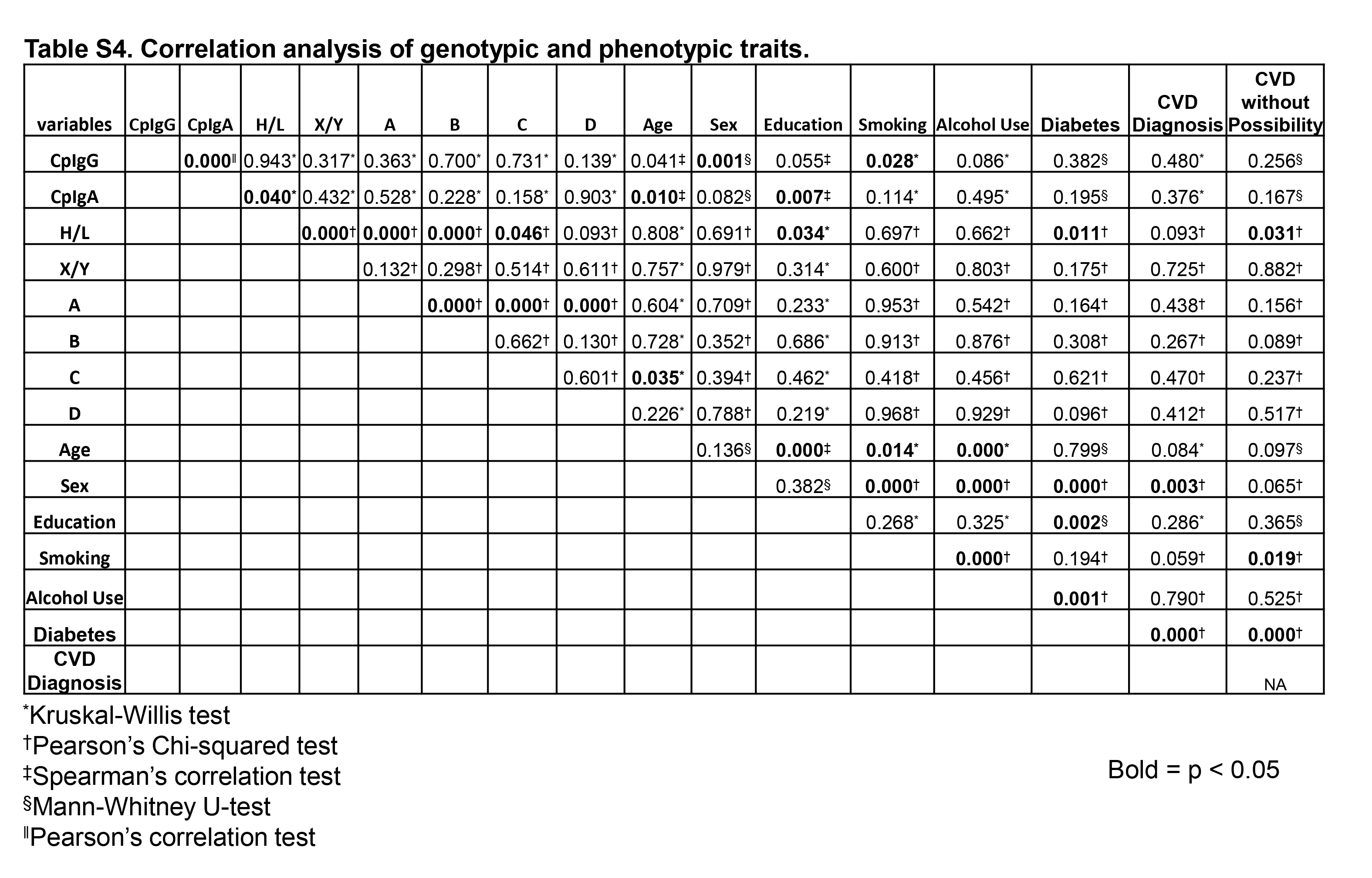

Supplement: S4 Table — Pairwise associations of traits were performed using a Spearman’s correlation analysis for all genetic polymorphisms assayed and a number of important covariates. Pink indicates p<0.05 and red indicates p<0.01. (TIF) [file pone.0210640.s006.tif]
